# Increased Carcinoembryonic Antigen (CEA) Level Is Highly Associated with Low Skeletal Muscle Mass in Asymptomatic Adults: A Population-Based Study

**DOI:** 10.3390/jcm11175009

**Published:** 2022-08-26

**Authors:** Chul-Hyun Park, Antonio Diaz Lizarraga, Yong-Taek Lee, Kyung-Jae Yoon, Tae-Kyung Yoo

**Affiliations:** 1 Department of Physical and Rehabilitation Medicine, Kangbuk Samsung Hospital, Sungkyunkwan University School of Medicine, 29 Saemunan-ro, Jongno-gu, Seoul 03181, Korea; 2 Department of Medicine, MetroWest Medical Center, 115 Lincoln St., Framingham, MA 01702, USA

**Keywords:** carcinoembryonic antigen, skeletal muscle mass, chronic inflammation, population study

## Abstract

We investigated the relationship between high carcinoembryonic antigen (CEA) levels and low skeletal muscle mass (LMM) in asymptomatic adults in a population-based study. A total of 202,602 adults (mean age 41.7 years) without malignancy, stroke, cardiovascular disease, or chronic lung/liver disease were included. A high CEA level was defined as ≥5 ng/mL. Skeletal muscle mass index (SMI) was calculated based on appendicular muscle mass (kg)/height (m)^2^. Participants were classified into three groups based on SMI: “normal”, “mild LMM”, and “severe LMM.” The prevalence of elevated CEA levels was the highest in subjects with severe LMM (4.2%), followed by those with mild LMM (1.6%) and normal muscle mass (1.1%) (*p* for trend < 0.001). In multivariate logistic regression analysis, high CEA was independently associated with having mild LMM (adjusted odds ratio, 1.139 [95% confidence interval, 1.092–1.188]) and severe LMM (2.611 [2.055–3.319]) compared to normal muscle mass. Furthermore, the association between high CEA and severe LMM was stronger in women than that in men (women, 5.373 [2.705–10.669]; men, 2.273 [1.762–2.933]). Elevated CEA levels were significantly associated with a higher prevalence of LMM. Therefore, increased CEA could be used as a biomarker for detecting LMM in adults without cancer.

## 1. Introduction

Carcinoembryonic antigen (CEA) is a fetal glycoprotein that is elevated in several pathologies [1]. Elevation in CEA level is a well-known prognostic serologic marker for colorectal cancer (CRC) and is associated with adenocarcinoma of the pancreas, lungs, prostate, ovaries, and breast [2]. Recently, multiple studies have reported its relationship with noncancerous, chronic inflammatory conditions such as chronic obstructive pulmonary disease (COPD), obesity, aging, and cigarette smoking [3,4,5,6]. In addition, CEA may be involved in chronic subclinical inflammation [3].

Low skeletal muscle mass (LMM) is a common condition throughout the continuum of care and is generally considered a predictor of poor outcomes; thus, it has clinically important implications [7]. It is also found in younger individuals with reduced muscle activity, ongoing physiological and pathological processes, and systemic inflammation [8,9]. Generally, muscle mass decreases by 3–8% every 10 years after 30 years of age due to changes in body composition and unfavorable metabolic alterations [10]. Due to this pathophysiology, LMM is considered a part of the aging process, with an estimated prevalence from 7% to over 50% in older people worldwide [11]. However, its prevalence can be high even among middle-aged and young populations, suggesting that LMM is prevalent in the general population, regardless of age [10].

CEA stimulates monocytes and macrophages and triggers the production of proinflammatory cytokines and endothelial adhesion molecules [12]. Positive correlations have been established between high-sensitivity C-reactive protein (hs-CRP), the neutrophil/lymphocyte ratio (NLR), and the CEA level [13]. Moreover, CEA and interleukin-6 (IL-6) levels have been positively associated, which is common in chronic inflammatory states [14,15]. Systemic inflammation may contribute to muscle breakdown, which leads to an enhanced inflammatory response [16]. However, to the best of our knowledge, no study has assessed the potential relationship between CEA levels and skeletal muscle mass in the general population without cancer. Therefore, we aimed to investigate the relationship between CEA levels and LMM in a healthy adult population.

## 2. Materials and Methods

### 2.1. Study Population

We included 249,441 Korean adults aged 18–89 years who underwent a comprehensive annual or biennial health check-up at Kangbuk Samsung Hospital Total Healthcare Centers in Seoul and Suwon, South Korea, between January 2012 and December 2018. This was a cross-sectional study using a subset of the Kangbuk Samsung Health Study (KSHS) [17,18]. KSHS is a cohort study of the Korean population who had an annual or biennial health check-up program. The purpose of this medical check-up program was to promote the health of participants by regular check-ups and to enhance early detection of existing diseases. Data were collected from the last-visit health examination data of the participant. The results of examinations, laboratory analysis, and responses to the standardized questionnaire from the health check-up of the participants were stored in the KSHS database. The data used in our study were extracted from the database.

**Figure 1:**
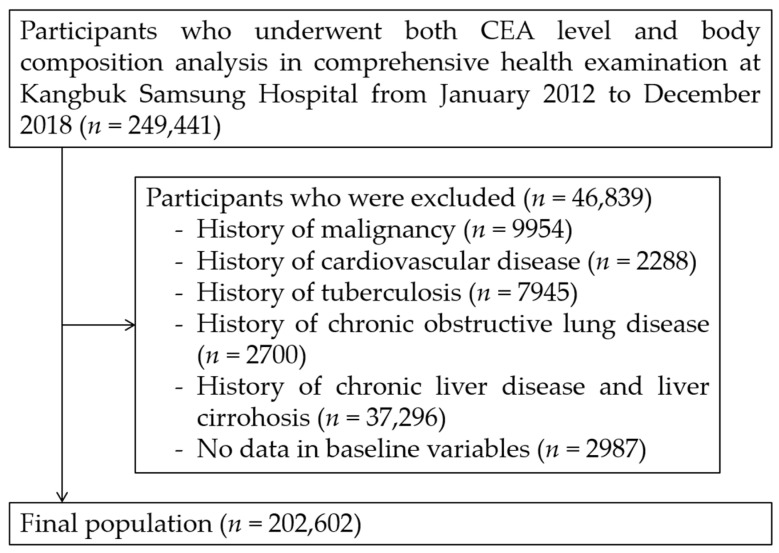
Selection of study population. CEA: carcinoembryonic antigen.

We excluded 46,839 participants based on the following criteria: histories of any malignancy (*n* = 9954), cardiovascular disease (*n* = 2288), tuberculosis (*n* = 7945), COPD (*n* = 2700), chronic liver disease/liver cirrhosis (*n* = 37,296), and missing baseline variables (*n* = 2987). Some individuals met more than one exclusion criteria. After exclusion (*n* = 46,839), 202,602 participants were included in the final analysis (Figure 1). The Institutional Review Board (IRB) of Kangbuk Samsung Hospital (IRB no. KBSMC 2022-02-030) approved our study protocol and waived the requirement for informed consent due to the use of de-identified datasets that were collected during the routine health check-up. Our study was performed in accordance with the ethical standards of the 1964 Declaration of Helsinki and its later amendments.

### 2.2. Data Collection and Classification of Participants

All data for this study were collected during a comprehensive health examination. The participants completed a standardized questionnaire providing information about their demographic characteristics, past medical history, and social history, including smoking and drinking status. Smoking status was defined as current smoker or non-smoker. Current smokers were defined as those who were smoking at the time of the health examination and had smoked >100 cigarettes in their lifetime, whereas those who had smoked more than 100 cigarettes in their lifetime but were not smoking at the time of the interview were categorized as former smokers. Participants who did not meet both criteria were classified as non-smokers [19]. Heavy alcohol consumption was defined by a consumption of >30 g of ethanol/day [20].

Trained medical personnel collected the following anthropometric measurements: height (cm) and weight (kg). BMI was defined as weight divided by height (m) squared. To estimate skeletal muscle mass, the appendicular skeletal muscle mass (kg) was measured using a bioelectrical impedance analyzer (BIA, InBody 720, Biospace, Seoul, Korea) [21]. To maintain the accuracy and consistency of the results, the BIA instrument was calibrated every morning before the initiation of the health examination. We calculated the skeletal muscle mass index (SMI) by dividing the appendicular skeletal muscle mass (kg) by the square of the height (m^2^) [22].

We classified the participants based on SMI according to the previous literature [23]. Participants with SMIs greater than −1 standard deviation (SD) of the sex-specific mean of young adults (age:18–39 years) were categorized as “normal”. Participants with SMIs within −1 to −2 SD (−2 < SD ≤ −1) and below −2 SD (SD ≤ −2) of the sex-specific mean of young adults were categorized as “mild LMM” and “severe LMM”, respectively. In this study population, sex-specific cut-off values for mild and severe LMM were 6.69 kg/m^2^ and 7.39 kg/m^2^ in men, and 5.44 kg/m^2^ and 4.70 kg/m^2^ in women, respectively.

Blood samples were collected after at least 8 h of fasting, and laboratory analyses were conducted for serum CEA, insulin, triglycerides (TG), total cholesterol, low-density lipoprotein cholesterol (LDL-C), high-density lipoprotein cholesterol (HDL-C), aspartate aminotransferase (AST), alanine aminotransferase (ALT), creatinine, and CRP levels. Serum CEA levels were measured using the following electrochemiluminescence immunoassay analyzers: Modular E170 (Roche Diagnostics, Tokyo, Japan) until April 2015, Cobas 8000 e602 (Roche Diagnostics), and Cobas 8000 e801 (Roche Diagnostics) from April 2015 to February 2018. To maintain quality control (QC), two levels of QC materials were run at least daily, or more frequently in case of changes that might impact the analytical results. In addition, prior to replacing the obsolete analyzer with a new one in the laboratory, its performance was evaluated to validate the measurement based on the Clinical and Laboratory Standards Institute guidelines [24,25,26].

The cut-off value for CEA was set at 5 ng/mL. CEA levels ≥ 5 ng/mL were defined as high CEA levels, whereas CEA levels < 5 ng/mL were defined as normal CEA levels based on a previous study [27]. All laboratory test results were confirmed by the Kangbuk Samsung Hospital Laboratory Medicine Department and validated by the Korean Association of Quality Assurance for Clinical Laboratories and the Korean Society of Laboratory Medicine [28].

### 2.3. Statistical Analysis

The chi-square test was used to compare categorical variables, and one-way analysis of variance (ANOVA) was used to compare continuous variables. Post hoc Bonferroni analyses were performed for group comparison. The prevalence (%) of high CEA levels in the normal, mild LMM, and severe LMM groups was compared using the chi-square test with post hoc analysis using the Bonferroni method. Furthermore, we used natural log-transformed (ln) CEA levels due to their positively skewed distribution, which was the best-fitting model for analysis, where the CEA level was treated as a continuous variable. Adjusted mean log-transformed CEA values between each group were compared using ANOCVA after adjusting for age, sex, history of hypertension, history of diabetes, and HDL-C, ALT, and CRP levels.

Multivariable logistic regression analyses were conducted to assess the association between high CEA levels, mild LMM and severe LMM. Three models adjusted for confounding factors were used. The adjustment for each model was as follows: model 1, adjusted for age, sex, and screening center; model 2, adjusted for model 1 plus smoking and drinking status; and model 3, adjusted for model 2 plus systolic blood pressure (SBP), serum insulin, TG, ALT, creatinine, and CRP.

Odds ratios (OR) were used to calculate the risk of mild and severe LMM compared with that in the normal group in participants with high CEA levels. Moreover, 95% confidence intervals (CI) were calculated. Subgroup analyses were performed using model 3 by stratifying the participants based on age (<40 years, 40–59 years and ≥60 years) and sex. For the statistical analysis, a two-tailed *p* value < 0.05 was considered significant. IBM SPSS version 26.0 (IBM Co., New York, NY, USA) was used for all the statistical analyses.

## 3. Results

### 3.1. Baseline Characteristics

Among the 202,602 study subjects, the mean age was 41.7 ± 9.4 years, and 49.5% were men (Table 1). The number of participants with normal skeletal muscle mass was 177,445; mild LMM, 22,800; and severe LMM, 2357. The mean SMIs were 7.2 ± 1.1 kg/m^2^ in the normal muscle mass group, 6.3 ± 0.9 kg/m^2^ in the mild LMM group, and 5.9 ± 0.8 kg/m^2^ in the severe LMM group. All the variables were significantly different between the groups, except for the percentage of screening center (*p* = 0.662) and total cholesterol (*p* = 0.428).

### 3.2. Comparison of CEA Levels between Subjects Classified by Skeletal Muscle Mass

**Figure 2:**
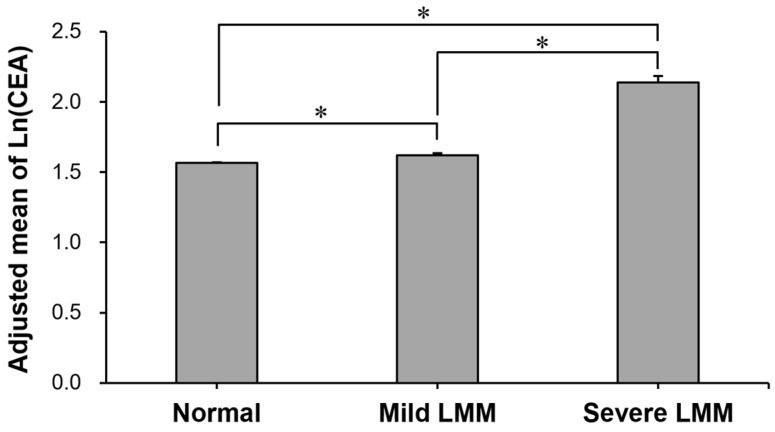
Comparison of adjusted mean of ln(CEA) between normal, mild LMM, and severe LMM groups. Adjusted means (±SE) of natural-log-transformed CEA levels in the groups were estimated from ANCOVA after adjustments for age, sex, history of hypertension, history of diabetes, HDL-C, ALT, and CRP. *: Group difference by Bonferroni post hoc *p* < 0.001. ALT: alanine aminotransferase; CEA: carcinoembryonic antigen; CRP: C-reactive protein; HDL-C: high-density lipoprotein cholesterol; LMM: low muscle mass; SE: standard error.

The prevalence of high CEA level according to skeletal muscle mass is compared in Table 2. It was the highest in individuals with severe LMM (4.2%), followed by those with mild LMM (1.6%) and normal muscle mass (1.1%) (*p* for trend < 0.001). The proportion (4.2%) in the severe LMM group was more than 3 times higher than in the normal muscle mass group (1.1%). After adjusting for possible confounding factors, the adjusted mean CEA was the highest in individuals with severe LMM, followed by those with mild LMM and normal muscle mass (*p* for trend < 0.001) (Figure 2). In the post hoc analysis, there were significant group differences in the adjusted mean CEA in all group comparisons (all post hoc Bonferroni *p* < 0.001, Figure 2).

### 3.3. Association between High CEA Levels and LMM

Table 3 shows the multivariable logistic regression analyses for the association between high CEA levels and LMM. In model 1 of the multivariate logistic analysis, a high CEA level was independently associated with mild LMM (adjusted odds ratio [aOR], 1.357; 95% CI, 1.212–1.519) and severe LMM (aOR, 2.669; 95% CI, 2.165–3.290), compared to normal muscle mass. In model 2, high CEA levels were consistently associated with mild LMM (model 2, aOR, 1.362; 95% CI, 1.207–1.537) and severe LMM (aOR, 2.820; 95% CI, 2.259–3.520) compared to normal muscle mass. In model 3, high CEA levels were independently associated with mild LMM (aOR, 1.139; 95% CI, 1.092–1.188) and severe LMM (aOR, 2.611; 95% CI, 2.055–3.319) compared to normal muscle mass.

### 3.4. Subgroup Analysis by Age and Sex

Subgroup analyses between high CEA and LMM were performed in groups stratified by age and sex (Table 4). In younger (<40 years), middle-aged (40~59 years) and elderly (≥60 years) subgroups, high CEA levels were significantly associated with severe LMM, respectively, showing the highest OR in the elderly group. There was a positive trend of increasing ORs from younger to middle-aged to elderly participants. In the subgroup analysis stratified by sex, high CEA levels and severe LMM were significantly associated in both men and women, and the association in women (aOR 5.373, 95% CI 2.705–10.669) was stronger than that in men (aOR 2.273, 95% CI 1.762–2.933).

## 4. Discussion

To the best of our knowledge, this is the first study to assess the relationship between high CEA levels and LMM in a population without cancer or other severe medical conditions. Our study showed that high CEA levels are strongly associated with decreased skeletal muscle mass. This result is consistent, even after adjusting for multiple confounding factors. Furthermore, the associations persisted in the subgroup analyses by age and sex. Interestingly, the association between high CEA levels and severe LMM is stronger in women than in men.

The association between high CEA levels and cancer is widely known in the medical community [29]. Previous studies have demonstrated that up to one-third of patients with CRC develop sarcopenia [30]. Despite the frequent coexistence of high CEA levels and LMM in the cancer population, no study has assessed whether this coexistence is mediated by cancer or the possibility of a direct relationship between CEA levels and low muscle mass.

CEA has been associated with several benign conditions, particularly chronic inflammation, and a previous study has investigated its association with widely accepted inflammatory markers, such as neutrophil/lymphocyte ratio, C-reactive protein, and IL-6 [12,13,14,15]. Proinflammatory cytokines and growth factors released as part of the systemic inflammatory response are known to participate in the muscle breakdown process [16]. After CEA is released from the gastrointestinal tract, it is primarily metabolized in the liver [31]. Animal studies have demonstrated the role of Kupffer cells in clearing CEA from circulation through the CEA receptor [12]. Simultaneously, this leads to activated macrophages capable of producing and releasing interleukin-1β (IL-1β), IL-6, and tumor necrosis factor alpha (TNF-α), which are known to contribute to muscle breakdown [12,32,33].

Previous experimental studies identified different mechanisms through which inflammatory molecules can participate in muscle breakdown. IL-6 facilitates muscle atrophy by blunting muscle anabolism through the activation of its intracellular receptor, leading to an increase in SOCS-3 mRNA, thereby resulting in the attenuation of growth hormone signaling [34]. IL-1β contributes to muscle degradation by reducing the amount of myofibrillar proteins in differentiated myotubes [32]. TNF-α is known to degrade mature muscles by accelerating protein degradation [33]. The elevated CEA levels found in our relatively young population with low muscle mass may be associated with a state of low-grade chronic inflammation. Multiple studies have shown that CEA is associated with inflammation, supporting our findings [3].

In the subgroup analysis by gender, there was a stronger association between CEA levels and low muscle mass in women than in men. There are several possible explanations for this observation. First, sex-specific hormones such as estrogen and testosterone can affect skeletal muscle mass. Estrogen plays a protective role in skeletal muscle by decreasing inflammation, and testosterone increases muscle protein anabolism and strength [35,36]. Therefore, these sex hormones may mediate the difference in the association between CEA levels and muscle mass. Second, the distribution and metabolic effects of skeletal musculature are somewhat different between men and women [37], which may affect the association between muscle mass and CEA levels. Furthermore, the present finding of sex differences is in line with that of our previous study. Previously, our team reported a higher association between high-sensitivity CRP (hs-CRP) and sarcopenic obesity in women than in men, which suggested that the association between inflammatory markers (e.g., hs-CRP) and adverse body composition (e.g., muscle and fat) is stronger in women [38]. Therefore, CEA, as a marker of inflammation, may have a stronger association in women than in men. In the subgroup analysis by age, the association between CEA levels and severe LMM was highest in elderly participants. A possible explanation is that a subclinical low-grade inflammation is increased with aging. Because CEA may play a role as an inflammatory marker, the association between CEA and LMM could be higher in elderly participants than younger participants.

There are some chronic diseases and conditions in which a high CEA level can be detected (≥5 ng/mL) (=false positive elevation). Chronic obstructive lung disease (COPD) is one of the known diseases that can be shown to have elevated CEA (or false elevation). In the previous study, the rate of high CEA level in COPD patients was 4.8% [4]. Compared to the previous study, the proportion of high CEA in this study was 4.2% in participants with severe LMM, which is similar to that in COPD patients (4.8%). In this study, participants with possible pathologies or diseases that can be associated with increased CEA level, such as malignancy, chronic lung/liver disease, and tuberculosis, were excluded. Therefore, we assume that the study participants are apparently healthy adults. The clinical implications of this study are as follows: First, severe LMM status should be considered as one of the conditions that can be detected with high CEA in participants without cancer. Second, a high CEA level could be regarded as not only a cancer marker but also an inflammation marker that can be increased in chronic inflammation diseases, such as COPD and low muscle mass related to sarcopenia. Therefore, increased CEA status could be utilized as a biomarker for low muscle mass or sarcopenia.

This study is unique in several respects. We incorporated a large number of cohorts (*n* = 202,602) in our study with a large number of events. Moreover, although previous studies mainly used geriatric populations to assess skeletal muscle mass and other biologic markers, our study participants were relatively young (mean age 41.7 ± 9.4), suggesting a new perspective in a relatively young population [39]. We adjusted for confounding factors known to be associated with LMM, which strengthened our study results [3,40]. In addition, we conducted a subgroup analysis to verify the relationship between both young and old age populations, which showed consistent results. Another strength of our study is that we excluded participants with histories of cancer and other conditions, such as malignancy, cardiovascular disease, tuberculosis, chronic obstructive lung disease, and liver cirrhosis, which can affect the relationship at baseline [41,42,43,44].

Despite these strengths, our study had several limitations. First, our study was a retrospective, cross-sectional study. Although we suggest a potential relationship, our study cannot validate a causal relationship. Second, our study participants were single-race, Korean, and relatively middle-aged, which may limit the generalizability of our study results to other age and ethnic groups. However, this is also a strength of our study, as our study participants were less affected by underlying comorbidities. Third, our study did not consider the physical activity level of the participants, which can affect muscle mass and CEA levels [45]. Future prospective studies including various age groups and ethnicities are required to verify the results of our study. Fourth, the possibility of late-developing cancer was not evaluated in this study. We tried to exclude severe existing diseases including malignancy. Nevertheless, the possibility of developing cancer or hidden malignancy in participants with high CEA needs to be considered in follow-up research. Fifth, indexes for skeletal muscle mass were not compared. There are several methods estimating indexes for skeletal muscle mass such as appendicular muscle mass dividing by height, body weight, or BMI, and whole-body muscle mass dividing by body weight [46]. Therefore, a future study should compare these indexes to find out the best index for determining low muscle mass that is highly associated with an increased CEA.

In conclusion, our study demonstrated that high CEA levels are significantly associated with decreased skeletal muscle mass. This relationship was consistent across the age and sex subgroups. In addition, the association between high CEA levels and low muscle mass was stronger in women than in men. Overall, our study results suggest that CEA levels could be a novel biomarker for LMM in healthy adults, especially women and older adults.

## Figures and Tables

**Table 1:** Baseline characteristics of study subjects classified by skeletal muscle mass.

	Total	Normal	Mild LMM	Severe LMM	* *p* Value
Number of subjects (*n*)	202,602	177,445	22,800	2357	
Age (years)	41.7 ± 9.4	41.7 ± 9.2	41.9 ± 10.8	44.7 ± 13.5	<0.001 ^†,‡,#^
Sex, Men	49.5	48.4	55.9	67.2	<0.001 ^†^
Screening center, Seoul	35.9	36.0	35.8	35.8	0.662 ^†^
Height (cm)	166.9 ± 8.6	167.2 ± 8.6	164.7 ± 8.1	163.3 ± 7.9	<0.001 ^†,‡,#^
BMI (kg/m^2^)	23.5 ± 3.5	23.9 ± 3.4	20.6 ± 2.2	19.1 ± 2.1	<0.001 ^†,‡,#^
Appendicular skeletal muscle mass (kg)	20.1 ± 4.9	20.5 ± 4.9	17.4 ± 4.0	15.9 ± 3.5	<0.001 ^†,‡,#^
SMI (kg/m^2^)	7.1 ± 1.1	7.2 ± 1.1	6.3 ± 0.9	5.9 ± 0.8	<0.001 ^†,‡,#^
Current smoker	13.8	13.6	15.0	19.4	<0.001 ^†^
Heavy drinking ^a^	18.0	18.1	17.2	19.2	<0.001 ^†^
Systolic blood pressure (mmHg)	109.6 ± 12.7	110.1 ± 12.7	106.5 ± 12.0	106.9 ± 12.8	<0.001 ^†,‡^
Diastolic blood pressure (mmHg)	70.4 ± 9.7	70.6 ± 9.7	69.3 ±9.3	69.9 ± 9.3	0.002 ^†,‡,#^
Hypertension	8.7	8.8	7.9	10.0	<0.001 ^†^
Diabetes mellitus	2.5	2.4	2.9	4.9	<0.001 ^†^
Insulin (mg/dL)	7.2 ± 4.7	7.4 ± 4.9	5.8 ± 3.4	5.1 ± 3.0	<0.001 ^†,‡,#^
Glucose (mg/dL)	96.9 ± 15.2	97.1 ± 15.0	95.8 ± 16.0	96.7 ± 19.9	<0.001 ^†,#^
Triglycerides (mg/dL)	114.1 ± 79.4	115.7 ± 81.2	102.8 ± 64.2	100.9 ± 60.5	<0.001 ^†,‡,#^
Total cholesterol (mg/dL)	190.8 ± 34.0	190.8 ± 33.9	190.8 ± 34.0	190.2 ± 35.9	0.428
LDL-C (mg/dL)	125.9 ±32.9	126.1±32.9	124.5±33.2	122.4 ±34.7	<0.001 ^†,‡,#^
HDL-C (mg/dL)	60.9 ± 16.2	60.4 ± 16.1	64.1 ± 16.3	65.5 ± 17.5	<0.001 ^†,‡,#^
AST (IU/L)	22.0 ± 14.0	22.1 ± 14.2	21.3 ± 11.0	23.4 ± 23.5	<0.001 ^†,‡,#^
ALT (IU/L)	23.2 ± 19.5	23.5 ± 20.0	20.6 ± 14.6	21.1 ± 19.1	<0.001 ^†,‡^
Creatinine (mg/dL)	0.82 ± 0.22	0.82 ± 0.22	0.81 ± 0.19	0.82 ± 0.27	<0.001 ^†^
CRP (mg/dL)	0.11 ± 0.30	0.12 ± 0.29	0.10 ± 0.31	0.13 ± 0.45	0.041 ^†,#^

Data are presented as mean ± SD, median (IQR), or percentage. * *p* values for between-group difference by one-way ANOVA in continuous variables or by χ^2^ test in categorical variables. Group comparisons by Bonferroni post hoc analysis were conducted after one-way ANOVA. ^†^: Bonferroni post hoc *p* < 0.05 for group comparison of normal vs. mild LMM ^‡^: Bonferroni post hoc *p* < 0.05 for group comparison of normal vs. severe LMM. ^#^: Bonferroni post hoc *p* < 0.05 for group comparison of mild LMM vs. severe LMM. ^a^ ≥ 20 g/day. ALT: alanine aminotransferase; AST: aspartate aminotransferase; BMI: body mass index; CRP: C-reactive protein; HDL-C: high-density lipoprotein cholesterol; LDL-C: low-density lipoprotein cholesterol; LMM: low muscle mass; SMI: skeletal muscle mass index. SMI (kg/m^2^) = appendicular skeletal muscle mass (kg)/height (m)^2^.

**Table 2:** Proportion of high CEA level for subjects classified by skeletal muscle mass (*n* = 202,602).

	Normal	Mild LMM	Severe LMM	*p* for Trend
Classification according to CEA level				<0.0001
Normal CEA level (<5 ng/mL) (%)	98.9	98.4	95.8	
High CEA level (≥5 ng/mL) (%)	1.1	1.6	4.2	

CEA: carcinoembryonic antigen; LMM: low muscle mass.

**Table 3:** Multivariate regression analyses showing associations of increased CEA with LMM.

	Mild LMM, OR (95% CI)	Severe LMM, OR (95% CI)
Model 1		
Normal (<5 ng/mL)	1 (reference)	1 (reference)
High CEA level (≥5 ng/mL)	1.357 (1.212–1.519)	2.669 (2.165–3.290)
Model 2		
Normal (<5 ng/mL)	1 (reference)	1 (reference)
High CEA level (≥5 ng/mL)	1.362 (1.207–1.537)	2.820 (2.259–3.520)
Model 3		
Normal (<5 ng/mL)	1 (reference)	1 (reference)
High CEA level (≥5 ng/mL)	1.139 (1.092–1.188)	2.611 (2.055–3.319)

ORs were calculated as the risks of having mild, low or severely low skeletal muscle mass according to the presence of high CEA level. Model 1: adjusted for age, sex, screening center. Model 2: adjusted for age, sex, screening center, smoking status, and heavy drinker. Model 3: adjusted for age, sex, screening center, smoking status, heavy drinker, SBP, insulin, triglycerides, ALT, creatinine, and CRP. ALT: alanine aminotransferase; CEA: carcinoembryonic antigen; CI: confidence interval; CRP: C-reactive protein; LMM: low muscle mass; OR: odds ratio; SBP: systolic blood pressure.

**Table 4:** Subgroup analyses by age and sex for associations of increased CEA with LMM.

	Mild LMM, OR (95% CI)	Severe LMM, OR (95% CI)
Age, years		
<40 (*n* = 95,817)	1.255 (0.975–1.452)	1.342 (1.301–1.398)
40~59 (*n*= 97,466)	1.047 (0.879–1.247)	2.283 (1.638–3.182)
≥60 (*n* = 9319)	1.609 (1.184–2.185)	3.149 (2.040–4.860)
Sex		
Men (*n* = 100,278)	1.261 (1.099–1.447)	2.273 (1.762–2.933)
Women (*n* = 102,324)	1.383 (0.933–2.049)	5.373 (2.705–10.669)

Adjusted ORs were calculated as the risks of having mild and severe low skeletal muscle mass (LMM) according to the presence of high CEA level in each subgroup after adjustments for age, sex, screening center, smoking status, heavy drinker status, SBP, insulin, triglycerides, ALT, creatinine, and CRP. ALT: alanine aminotransferase; CEA: carcinoembryonic antigen; CI: confidence interval; CRP: C-reactive protein; LMM: low muscle mass; OR: odds ratio; SBP: systolic blood pressure.

## Data Availability

Data can be obtained from the corresponding author upon reasonable request.
